# Supplementary material for: Identification of photoperiod-induced specific miRNAs in the adrenal glands of Sunite sheep (Ovis aries)
Source: Front Vet Sci. 2022 Jul 22;9:888207. doi: 10.3389/fvets.2022.888207 (PMC9354845; doi:10.3389/fvets.2022.888207)
Supplement: Supplementary file 1 [file Data_Sheet_1.ZIP › Supplementary file/miRDeep2 file.zip!/miRDeep2 file/Know_Structure/SPLP42A3/oar-mir-106b.pdf]

miRBase precursor : oar-mir-106b
Total read count : 2659
oar-miR-106b read count : 741
remaining reads : 1918

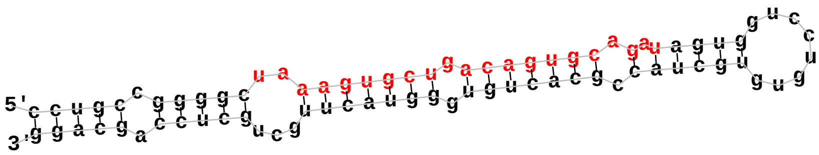

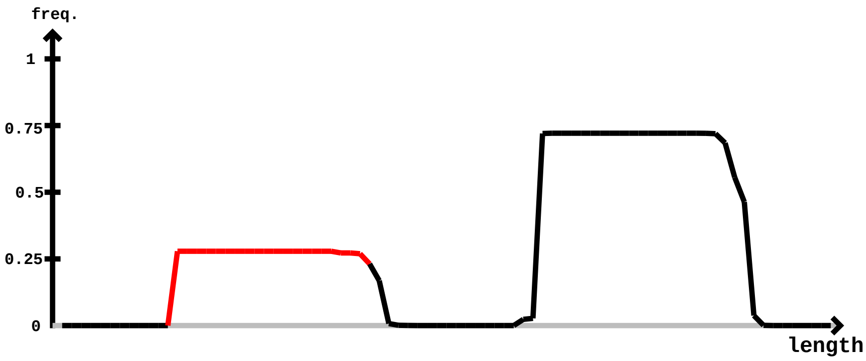


```
              oar-miR-106b
5'- ccugccgggcuaaagugcugacagugcagauaguggucccugugugcuaccgcacuguggguacuugcugcuccagcagg   -3'    exp
    (((((.(((((..(((((((.(((((((.(.(((((.......)))))).))))))).)))))))...))))).)))))    reads      mm         sample
    ..........uaaagugcugacagugc....................................................    14         0          seq
    ..........uaaagugcugacagugA....................................................    3          1          seq
    ..........uaaagugcugacagugcag..................................................    8          0          seq
    ..........uaaagugcugacagugcaga.................................................    101        0          seq
    ..........uaaagugcugacagugcagau................................................    167        0          seq
    ..........uaaagugcugacagugcagaua...............................................    428        0          seq
    ..........uaaagugcugacagugcagauag..............................................    17         0          seq
    ..........uaaagugcugacagugcagauagu.............................................    2          0          seq
    ..........uaaagugcugacagugcagauagug............................................    1          0          seq
    ..............................................uaccgcacuguggguacuu..............    1          0          seq
    ..............................................uaccgcacuguggguacuugc............    15         0          seq
    ..............................................uaccgcacuguggguacuugcu...........    45         0          seq
    ..............................................uaccgcacuguggguacuugcug..........    1          0          seq
    ...............................................accgcacuguggguacuugc............    2          0          seq
    ...............................................accgcacuguggguacuugcu...........    4          0          seq
    ...............................................accgcacuguggguacuugcug..........    2          0          seq
    ...............................................accgcacuguggguacuugcugc.........    2          0          seq
    ................................................ccgcacuguggguacuug.............    4          0          seq
    ................................................ccgcacuguggguacuugc............    75         0          seq
    ................................................ccgcacuguggguacuugcu...........    292        0          seq
    ................................................ccgcacuguggguacuugcug..........    242        0          seq
    ................................................ccgcacuguggguacuugcugc.........    1130       0          seq
    ................................................ccgcacuguggguacuugcugcu........    98         0          seq
    ................................................ccgcacuguggguacuugcugcuc.......    2          0          seq
    .................................................cgcacuguggguacuugcu...........    2          0          seq
    .................................................cgcacuguggguacuugcugc.........    1          0          seq
```
